# Optimization and Mathematical Modeling of Biocompound Extraction from *Pterocaulon* Species Using Deep Eutectic Solvents

**DOI:** 10.3390/molecules31213849

**Published:** 2026-10-25

**Authors:** Victor Mateus Juchem Salerno, Henrique Martins Tavares, Victor Hugo Silva Rodrigues, Bruna Medeiros-Neves, Helder Ferreira Teixeira, Gabriela de Carvalho Meirelles, Gilsane Lino von Poser, Eduardo Cassel, Rubem Mário Figueiró Vargas

**Affiliations:** 1 Laboratório de Operações Unitárias, Escola Politécnica, Pontifícia Universidade Católica do Rio Grande do Sul, Porto Alegre 90619-900, RS, Brazil; 2 Programa de Pós-graduação em Ciências Farmacêuticas, Universidade Federal do Rio Grande do Sul, Porto Alegre 90610-000, RS, Brazil

**Keywords:** deep eutectic solvent, *Pterocaulon*, mass transfer, mathematical modelling, biocompound

## Abstract

Species from the genus *Pterocaulon* are recognized for their pharmacological relevance attributed to the presence of coumarins and phenolic compounds such as flavonoids and phenolic acids. This study aimed to optimize the extraction of these compounds using deep eutectic solvents (DES) via a Box–Behnken experimental design. For the *Pterocaulon* species (*P. Polystachyum*, *P. balansae*, and *P. lorentzii*), extraction time, ultrasonic power, and ultrasonic probe pretreatment cycles were evaluated. The regression models obtained showed good fits for *P. lorentzii* (R^2^ = 96.09%), whereas for *P. polystachyum* and *P. balansae*, the models were less predictive, reflecting the influence of the specific characteristics of each plant matrix. Optimal extraction conditions within the investigated range varied among the species, notably involving maximum ultrasonic power (275 W) and a 90 min extraction time for the *Pterocaulon* species studied, at a fixed temperature of 40 °C and a solid-to-liquid ratio of 0.05 (g/mL). Total phenolic content (TPC) was quantified using UV-Vis spectrophotometry. The chromatographic analysis (UFLC-DAD) showed the presence of the coumarins previously reported for the species under study, which were tentatively identified based on their retention times and UV absorption spectra. The main compounds tentatively identified were sabandinol in *P. lorentzii*; prenyletin-methyl-ether and 5-methoxy-6,7-methylenedioxycoumarin in *P. polystachyum*; and 5,6-dimethoxy-7-(2′,3′-epoxy-3-methylbutyloxy)coumarin, followed by 5-methoxy-6,7-methylenedioxycoumarin in *P. balansae*. In the extracts of the three species, compounds with the UV profile of phenolic acids were detected. The extraction kinetics were adequately described (R^2^ = 0.954–0.960 for *P. balansae*, 0.953 for *P. lorentzii*, and 0.918–0.923 for *P. polystachyum*) by a diffusion model with time-varying boundary conditions associated with the interfacial condition represented by different types of adsorption isotherms. These findings suggest that ultrasound-assisted extraction using DES (betaine:ethylene glycol 1:3) is an efficient and sustainable strategy for obtaining extracts rich in bioactive compounds of potential pharmaceutical and cosmetic interest.

## 1. Introduction

The genus *Pterocaulon* (Asteraceae) encompasses 26 species, some of which have been traditionally used for treating skin conditions and hepatic ailments [1]. The genus occurs predominantly in southern Brazil, Paraguay, northeastern Argentina, and Uruguay [2]. Species such as *Pterocaulon polystachyum*, *P. lorentzii*, and *P. balansae* have been reported to contain coumarins, which are considered important secondary metabolites associated with the pharmacological properties of the genus [1,3,4]. Other classes of compounds, including flavonoids, terpenes, phenolic acids, and polyacetylenes, have also been identified in *Pterocaulon* species [1].

Notable coumarins reported in these *Pterocaulon* species include 7-(2,3-epoxy-3-methylbutyloxy)-6-methoxycoumarin, 5-(2,3-epoxy-3-methylbutyloxy)-6,7-methylenedioxycoumarin, 7-(3-methyl-2-butenyloxy)-6-methoxycoumarin, and 5-methoxy-6,7-methylenedioxycoumarin [1,3,4]. Among these compounds, 5-methoxy-6,7-methylenedioxycoumarin has demonstrated cytotoxic effects against glioma and leukemia cells [5,6], whereas 7-(3-methyl-2-butenyloxy)-6-methoxycoumarin, also known as prenyletin methyl ether, has exhibited antifungal activity [1]. Furthermore, a mixture of these two coumarins has been reported to reduce the viability of T24 bladder cancer cells at concentrations ranging from 6 to 12 μg/mL [7].

Extracts from species such as *P. polystachyum*, *P. balansae,* and *P. lorentzii* have been obtained using conventional extraction techniques, including Soxhlet extraction [8,9], maceration [1,4], and percolation [10]. Although recognized for their high extraction capacity, these methods employ organic solvents such as methanol, hexane, and dichloromethane and exhibit low selectivity for the compounds of interest. Furthermore, given that the pharmaceutical market seeks products obtained through processes that adhere to green chemistry principles, the use of solvents that are often harmful to human health and the environment has driven the search for more sustainable alternatives.

In this context, green extraction techniques are widely investigated. Among them, the use of supercritical fluids has been studied to obtain extracts from *Pterocaulon* species [7,11,12]. Solid–liquid extraction using deep eutectic solvents (DES) has also shown the ability to remove bioactive molecules from these plant species [13,14], indicating the viability of these solvents for the extraction of compounds intended for pharmaceutical and nutraceutical applications. DES-based extraction operates at low temperatures and can be environmentally friendly and offer safe applications, depending on the HBA and HBD used [13], thereby aligning with the demand for processes with a green approach. DES-based extraction depends on several variables, such as temperature, extraction time, solid-to-liquid ratio, particle size, and solvent selectivity. Therefore, experimental designs that aid in understanding and optimizing the response of a system or extraction process involving deep eutectic solvents have been increasingly employed [15,16,17,18].

In the specific case of *P. polystachyum*, Rodrigues et al. [13] obtained extracts using a simple, green process, aiming for the application of these extracts, rich in phenolic compounds and coumarins. By comparing different extraction methods, they demonstrated the advantages of ultrasound-assisted extraction (UAE), which increased the efficiency of phenolic compound extraction. UAE can be applied indirectly using ultrasonic baths, which promote sample agitation and facilitate the release of intracellular compounds [19,20]; and using ultrasonic probes applied directly to the samples as pretreatment [21], which can also serve as a technique for direct cell disruption [21,22,23]. The authors also showed that extracts obtained using a DES composed of betaine and ethylene glycol in a 1:3 molar ratio exhibited the highest total phenolic content (TPC), surpassing traditional solvents used for comparison, such as water and ethanol. The DES extract demonstrated potential for use in cosmetic formulations, representing a novel, green, cost-effective, simple, and safe product that can be used without purification.

In another study, Salerno et al. [24] employed similar techniques, using computational modeling enabled by COSMO-RS in the context of DES to evaluate the extraction of benzopyrans from *Hypericum polyanthemum* Klotzsch ex Reichardt (Hypericaceae). The authors evaluated 10 mixtures computationally and selected the DES composed of lactic acid, glycine, and water (3:1:3 molar ratio) with HP1, HP2, and HP3 molecules, based on their σ-profiles. The results showed how DES outperformed organic solvents. Furthermore, the authors performed a mathematical simulation of the extraction process by describing its kinetics using a Crank diffusion model [25] adapted to represent mass transfer to the selected DES (R^2^ > 0.98; mean percentage error < 9%); this indicated diffusion-controlled kinetics and allowed for the estimation of effective diffusion coefficients.

Beyond process optimization, mechanistic mathematical modeling provides a useful framework for interpreting solid–liquid extraction kinetics by relating experimental extraction curves to the underlying mass-transfer mechanisms [26,27]. Diffusion-based models derived from Fick’s second law have been widely applied to plant matrices [24,25,26,27], particularly when the rapid washing of readily accessible solutes is followed by a slower intraparticle diffusion stage [26,28,29]. In batch systems with a finite solvent volume, intraparticle diffusion can be coupled to a mass balance in the well-mixed liquid phase, resulting in a time-dependent interfacial condition [15,24]; equilibrium relations may also be incorporated to describe solute partitioning between the solid and liquid phases. Such approaches allow the estimation of effective diffusion coefficients and provide useful information for process interpretation, optimization, and scale-up [24,26,27,30].

In this context, this study was designed to evaluate and optimize the total phenolic content of the DES extracts from the aerial parts of *P. polystachyum*, *P. balansae*, and *P. lorentzii* by investigating extraction time and the application of ultrasound (probe) as a pre-treatment, combined with UAE. We hypothesized that these parameters would affect extraction efficiency and that their optimization would improve compound recovery. The obtained extracts were evaluated for total phenolic content (TPC) as the response for optimization and extraction curve experiments. These curves were analyzed using a mathematical model based on the hypothesis of diffusive transport within the plant material when subjected to batch extraction. Under these conditions, the extract content in the solvent varies with time, combined with the interfacial description linked to different equilibrium relations based on adsorption isotherms. This is an innovative approach to evaluate DES extraction. Additionally, given the biological potential of coumarins among phenolic compounds, and in line with current trends toward the development of sustainable technologies, extracts were also analyzed for coumarin presence through chromatography qualitative analysis.

## 2. Results and Discussion

### 2.1. Extraction Optimization 

Following the operational planning phase, the extraction procedures were systematically executed according to the Box–Behnken design (Table 1). Each order represents one single extraction. The resulting extracts were subsequently quantified for total phenolic content (Table 1), generating the experimental data necessary for statistical modeling. These TPC values served as the response variable for the regression analysis performed in Minitab^®^ 19, allowing the evaluation of the main effects and interactions of the independent variables. Overall, *P. polystachyum* exhibited the highest amount of phenolic compounds at all the conditions studied, followed by *P. balansae* and *P. lorentzii*.

In the regression equations, the independent variables for the *Pterocaulon* design are represented by: TE (extraction time), PT (power), and NC (number of ultrasonic pretreatment cycles), with the results of TPC as the response variable. The models are presented in Table 2.

The distinct performance of the models reflects intrinsic differences in the chemical composition and plant matrix of each species. While *P. lorentzii* yielded a statistically significant model (R^2^ = 96.09%; p_model_ = 0.005), *P. polystachyum* and *P. balansae* exhibited responses that the quadratic model failed to capture adequately, suggesting the possible influence of unaccounted factors or the need for alternative modeling approaches.

Statistical indicators for the generated response surface models are presented in Table 2. In addition to R^2^, the overall model F- and *p*-values (F_model_ and p_model_), adjusted R^2^ (R^2^_adj_), predicted R^2^ (R^2^_pred_), coefficient of variation (CV), RMSE, and lack-of-fit *p*-value (p_LOF_) are reported for each plant matrix. The complete ANOVA tables, including the significance of the linear, quadratic, and interaction terms, residual error, pure error, lack of fit, and the corresponding degrees of freedom, are provided in the Supplementary Information. The coefficients of determination (R^2^) ranged from 70.89% to 96.09%; an R^2^ > 70% is considered acceptable, given the level of biological and environmental noise associated with the raw material used for extraction [31,32]. In addition to the coefficient of determination, the lack-of-fit *p*-value was considered. No statistically significant lack of fit was detected for any of the three plant matrices, with *p*-values of 0.277, 0.852, and 0.565 for *P. polystachyum*, *P. balansae*, and *P. lorentzii*, respectively. The root mean square error (RMSE) was also considered. The RMSE results for the three *Pterocaulon* species were: for *P. polystachyum*, RMSE = 2.74 (TPC values between 18.6 and 34.6); for *P. balansae*, RMSE = 2.72 (TPC between 6.4 and 24.5); and for *P. lorentzii*, RMSE = 0.206 (TPC between 1.6 and 6.0). The reported values indicate an acceptable magnitude of error, considering the order of magnitude of the TPC results obtained for the investigated plant species. However, a nonsignificant lack-of-fit test alone is not sufficient to demonstrate model adequacy. For *P. polystachyum*, the model presented R^2^ = 74.67%, R^2^_adj_ = 29.07%, R^2^_pred_ = 0.00%, CV = 18.77%, and an overall model *p*-value of 0.305 (F_model_ = 1.64), with a nonsignificant lack-of-fit test (p_LOF_ = 0.277). For *P. balansae*, the corresponding values were R^2^ = 70.89%, R^2^_adj_ = 18.49%, R^2^_pred_ = 0.00%, CV = 34.69%, and an overall model *p*-value of 0.387 (F_model_ = 1.35), with a nonsignificant lack-of-fit test (p_LOF_ = 0.852); the high CV obtained for this species is consistent with the relatively high variability observed at the center points. These results indicate that, although no statistically significant lack of fit was detected, the global quadratic models for these two species present limited explanatory and predictive performance and should therefore be interpreted cautiously. In contrast, the model for *P. lorentzii* showed stronger statistical performance, with R^2^ = 96.09%, R^2^_adj_ = 89.06%, R^2^_pred_ = 60.38%, CV = 9.62%, and an overall model *p*-value of 0.005 (F_model_ = 13.66), while the lack of fit remained nonsignificant (p_LOF_ = 0.565).

According to Anticona et al. [33], in addition to operational parameters, the geographical origin, species, and phytochemical profile of the plant are crucial factors impacting extract yield and characteristics. In this context, the experimental approach, using a probe sonicator as a pretreatment for *Pterocaulon* species, demonstrated that, although the principle of enhancing bioactive compound recovery via shear forces is theoretically sound, its practical efficacy is strongly modulated by the specific characteristics of each plant matrix. The inconsistency of the models for *P. polystachyum* and *P. balansae* highlights that the mere application of ultrasonic energy, without considering the structural and chemical particularities of each species, may be insufficient to ensure efficient extraction.

**Figure 1:**
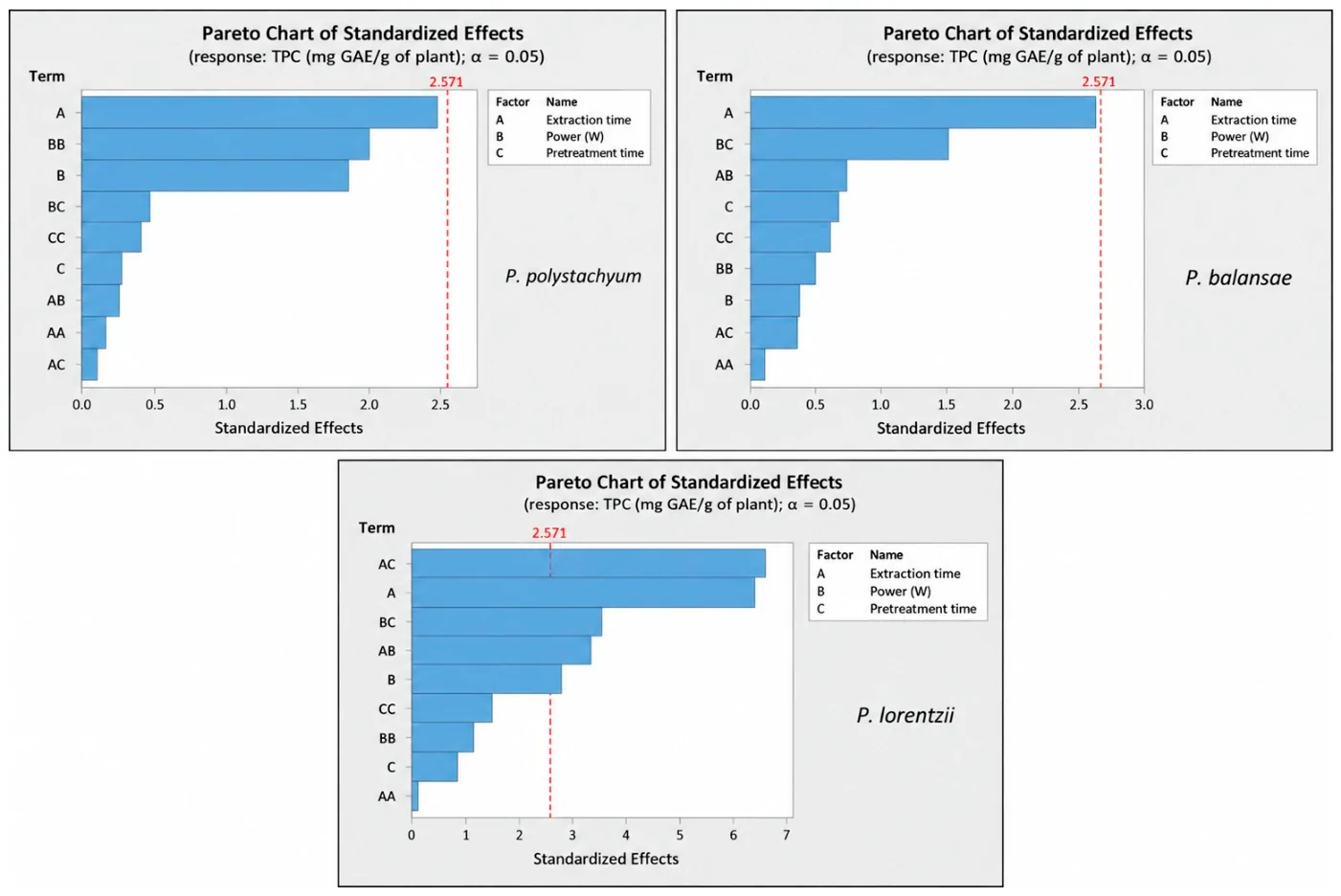
Pareto chart: standardized effect of the process variables considered in the extractions from *Pterocaulon* species (α = 0.05). Red dashed lines are related to the z-score (confidence interval).

Analysis of the results—derived from Pareto charts of standardized effects alongside ANOVA evaluations for each plant (Figure 1)—revealed a clear disparity in process efficacy among the investigated species; this corroborates and expands upon existing literature suggesting that the ultrasound mechanism of action can vary significantly depending on the plant source.

**Figure 2:**
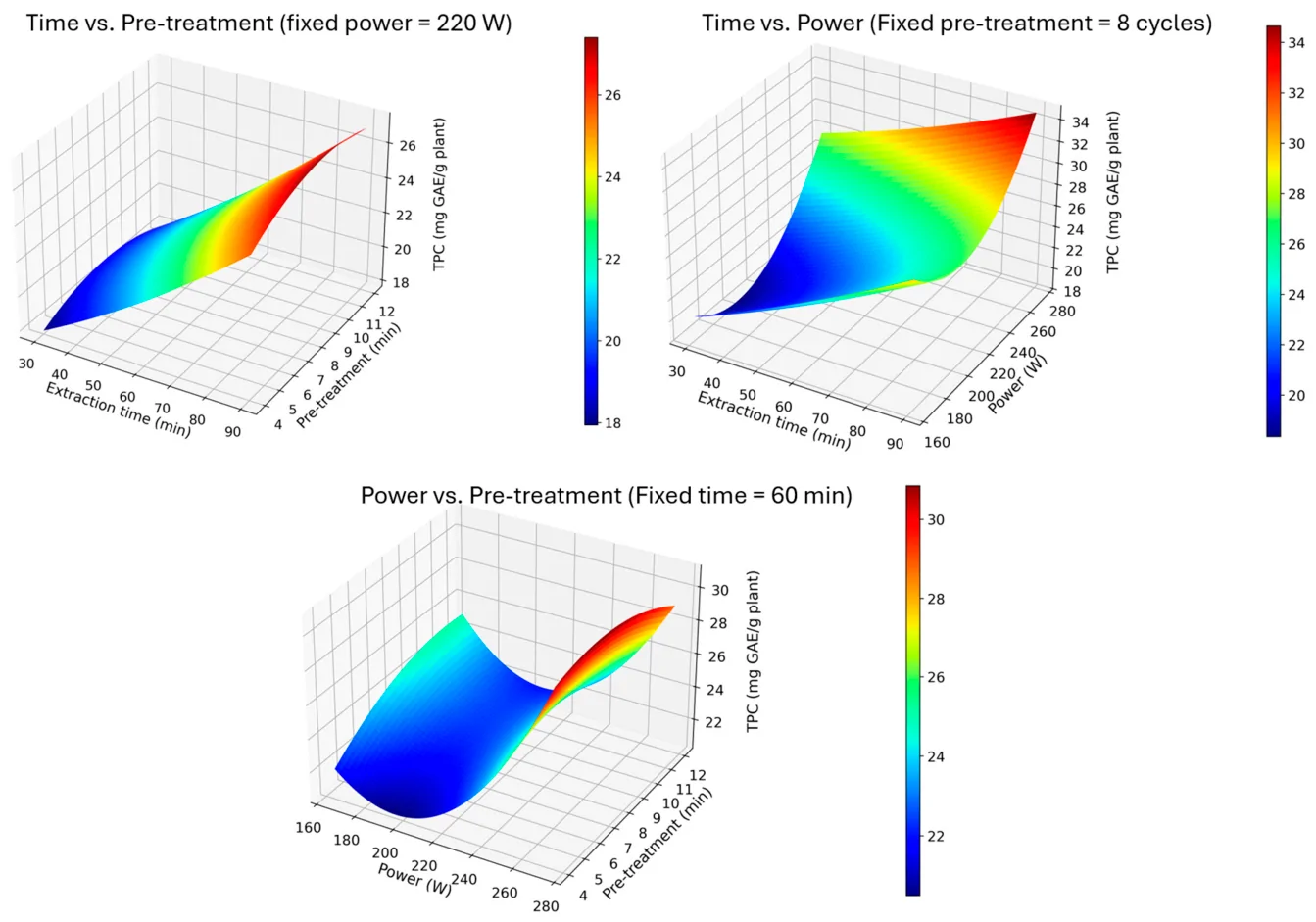
Estimated surface response for recovery of total phenolic content during the *P. polystachyum* extraction process.

**Figure 3:**
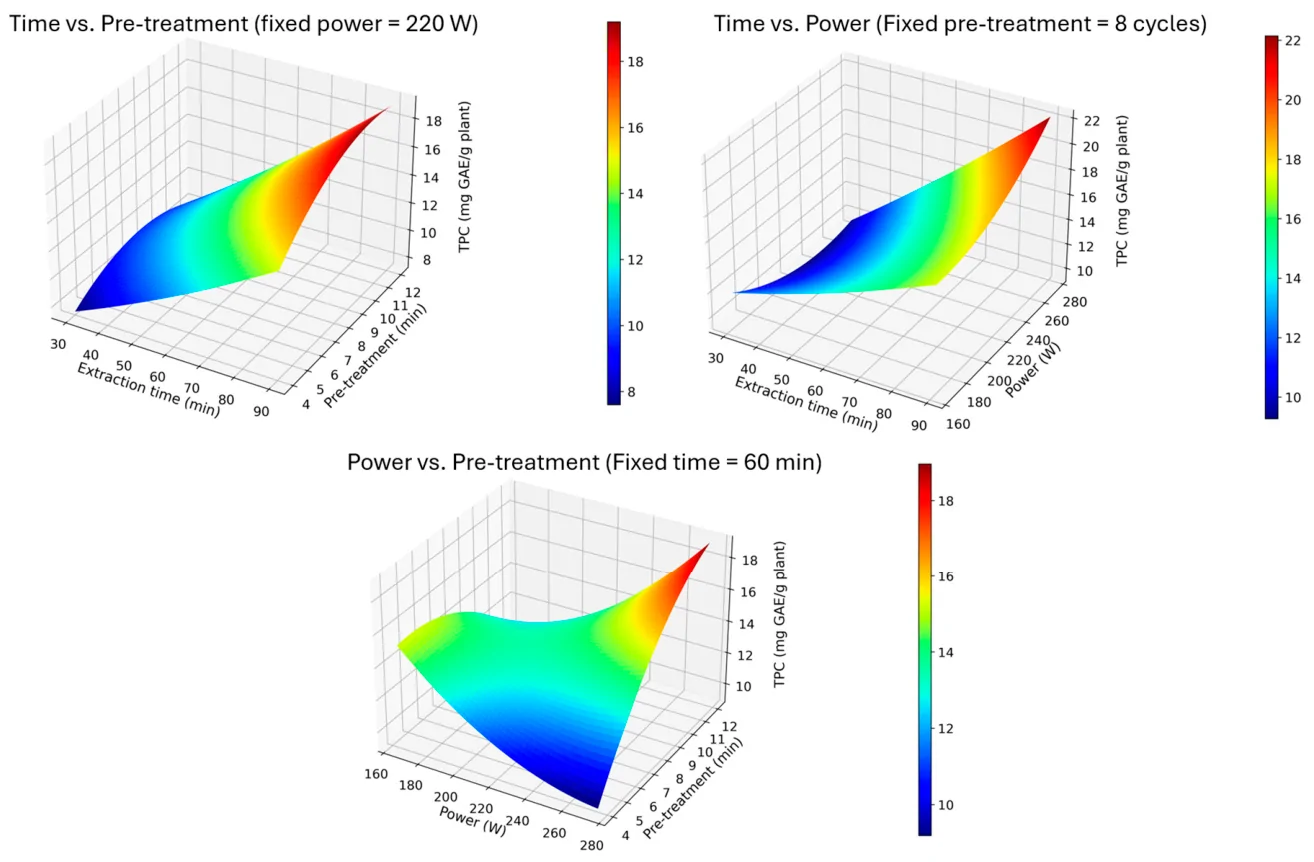
Estimated surface response for recovery of total phenolic content during the *P. balansae* extraction process.

**Figure 4:**
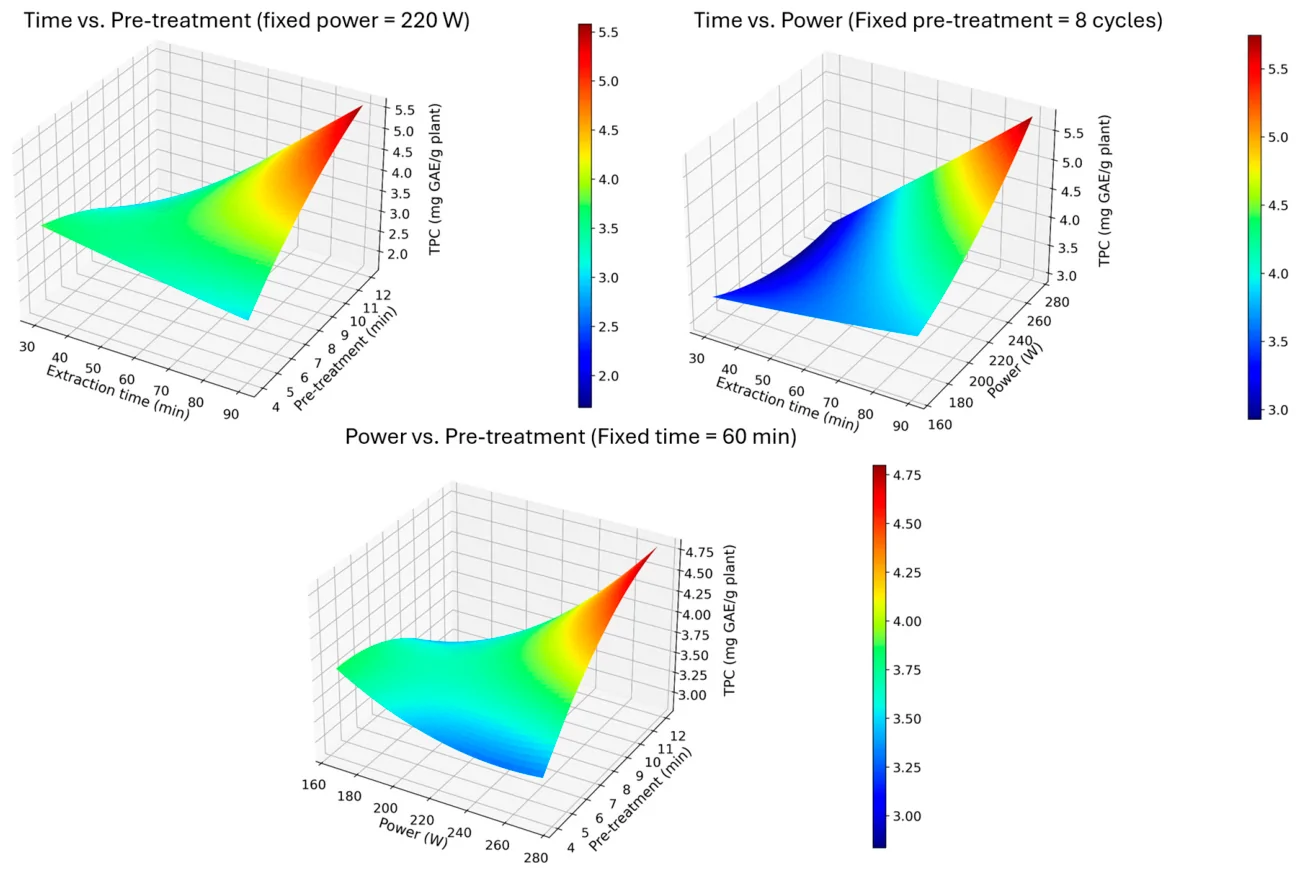
Estimated surface response for recovery of total phenolic content during the *P. lorentzii* extraction process.

Conducting the experiments was essential for a critical assessment of the process, even in cases where the models showed low statistical significance. The divergent responses observed across species—ranging from a robust model for *P. lorentzii* to models with lower predictive power regarding the chosen independent variables for *P. polystachyum* and *P. balansae*—demonstrated experimentally that the efficacy of ultrasound parameters is intrinsically dependent on the plant matrix. Thus, the collected data validated the predictions of the statistically significant models while simultaneously highlighting the design’s limitations for species whose extraction was not satisfactorily described by the tested variables, thereby enriching the analysis with concrete evidence regarding process specificity. Based on the regression model resulting from the application of the Box–Behnken design, the response surfaces are presented below (Figure 2, Figure 3 and Figure 4).

Response surface analysis for the *Pterocaulon* species indicates that maximum TPC may lie beyond the limits tested in the experimental design. The models suggest that using pretreatment times and sonication powers higher than those evaluated would potentially increase yield. In contrast, for *P. lorentzii*, where the Box–Behnken model identified the tested variables as the most influential, the maximum extraction point fell within the studied region.

Using the optimization tool in Minitab^®^ software, optimal extraction conditions within the investigated range were determined for each species investigated. For the *Pterocaulon* genus, a convergence in ultrasonic energy parameters was observed: all three species achieved maximum extraction efficiency with an extraction time (TE) of 90 min and a maximum power of 275 W. Pretreatment time (NC), however, varied among the species, with optimal values of 7.15 cycles for *P. polystachyum* and 12 cycles for *P. balansae* and *P. lorentzii*. Under these conditions, the predicted total phenolic content (TPC) values were 35.17, 26.51, and 7.40 mg GAE/g of plant material, respectively. These data align with the behavior observed in the response surfaces and reinforce the premise that optimal extraction conditions are highly specific to each plant matrix. These values were used to generate the respective extraction curves presented in Section 2.1.

#### Extraction with Deep Eutectic Solvents

Prior to extraction, the plant water content was determined after drying; the plant material was processed and then sieved to determine the mean particle diameter, and plant density was determined via gas pycnometry (data summarized in Table 3).

**Figure 5:**
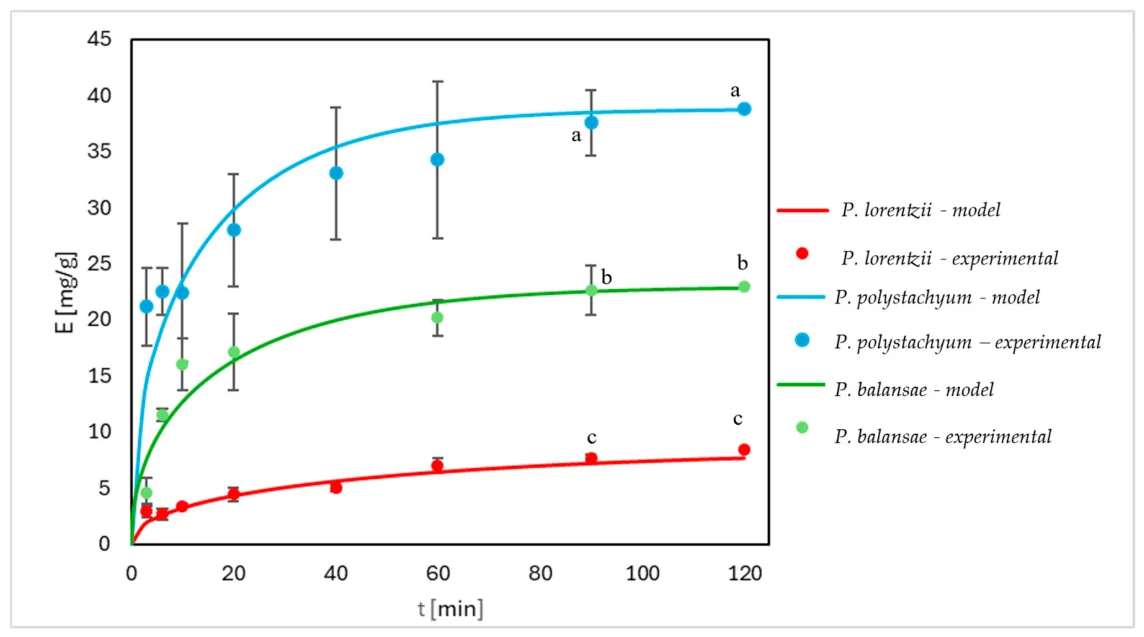
Plot of quantified sampling points for TPC and the mathematical model curve for *Pterocaulon* species. Different lowercase letters indicate statistically significant differences (*p* < 0.05) between TPC results. All points show standard deviation.

The extraction curves are presented in Figure 5. First, a spectrophotometric calibration curve was obtained to determine total phenolic content (TPC). The correlation showed linear behavior, described by the equation y (ABS) = 0.8425x (g GAE/L) − 0.0409, with R^2^ = 0.983. Subsequently, DES extraction curves with betaine:ethylene glycol 1:3 were performed (in triplicate), and the extracts were analyzed for TPC. These extractions were performed using the optimal conditions obtained within the tested range over a total duration of 120 min for the *Pterocaulon* genus. For *P. polystachyum* and *P. lorentzii*, samples were collected at 0, 3, 6, 10, 20, 40, 60, 90, and 120 min (N = 9). For *P. balansae*, samples were collected at 0, 3, 6, 10, 20, 60, 90, and 120 min (N = 8). These numbers of experimental observations were subsequently used in the calculation of the corrected Akaike Information Criterion. For *P. polystachyum*, the number of pretreatment cycles was rounded to 7. The highest TPC values were observed at 90 min (Figure 5). All *Pterocaulon* species reached their maximum concentration yield (mg GAE/g of plant material) at this time point; *P. polystachyum* showed the highest value (37.56 ± 7.01 mg GAE/g), followed by *P. balansae* (22.63 ± 1.60 mg GAE/g) and *P. lorentzii* (7.69 ± 0.67 mg GAE/g). Additionally, a statistical analysis comparing the TPC values obtained at 90 and 120 min showed no significant difference between the results, according to Tukey’s test. This supports the hypothesis that extraction appears to approach a plateau for these compounds or is at least very close to doing so.

**Figure 6:**
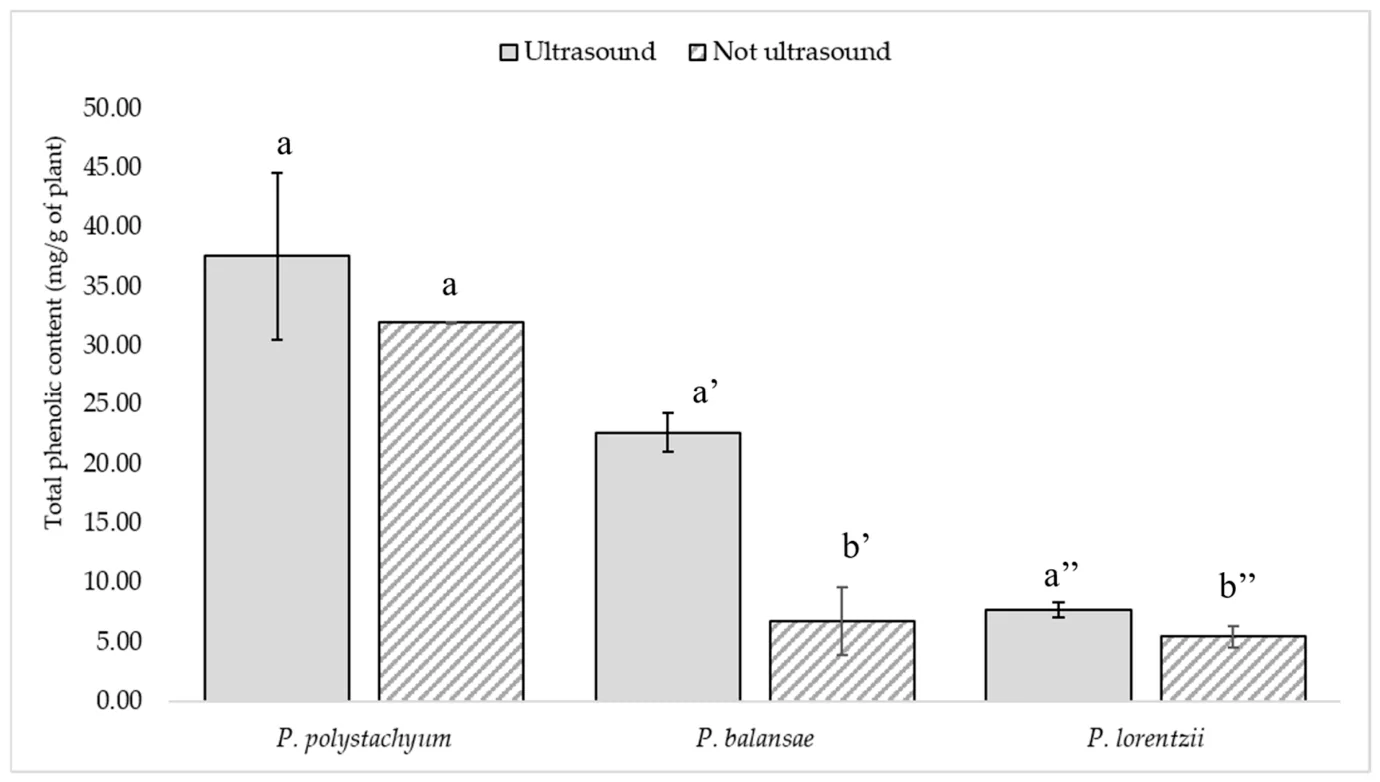
Comparative graph of the difference in extraction yield with DES using ultrasonication as a pretreatment versus without its application. Different lowercase letters indicate statistically significant differences (*p* < 0.05) in the TPC concentration obtained. Standard deviation bars representing the standard error are included for all data points; however, in some cases they are too small to be visible.

Finally, an extraction (triplicate) without probe pretreatment, under the same temperature and time conditions, yielded 32.01 ± 0.01, 6.75 ± 2.82, and 5.44 ± 0.93 mg GAE/g for *P. polystachyum*, *P. balansae*, and *P. lorentzii*, respectively (Figure 6). These results reinforce the Pareto analysis (Figure 1) and show that the effect of pretreatment is species-dependent: for *P. polystachyum*, no term was significant, which is consistent with the small gain; for *P. lorentzii*, the pretreatment acted mainly through interactions with extraction time and power; and for *P. balansae*, although only extraction time was significant in the Pareto chart, the with/without pretreatment comparison revealed a statistically significant increase in the TPC value (from 6.75 ± 2.82 to 22.63 ± 1.60 mg GAE/g), as confirmed by the Tukey test, indicating that the presence of the pretreatment step is more relevant than its duration within the tested range.

However, this result should be interpreted with caution, since the center points of *P. balansae* showed considerable variability (16.17, 12.55, and 6.48 mg GAE/g), reflecting the heterogeneity of the plant matrix and the limited repeatability of the model for this species. Therefore, the optimization of conditions for *P. balansae* should be considered exploratory, and validation in independent experiments, with new batches of plant material and a larger number of center-point replicates, is recommended before any practical application. Nevertheless, the results presented in Figure 6 help to confirm the optimal condition found within the range tested.

### 2.2. Coumarin Identification in the Extracts

**Figure 7:**
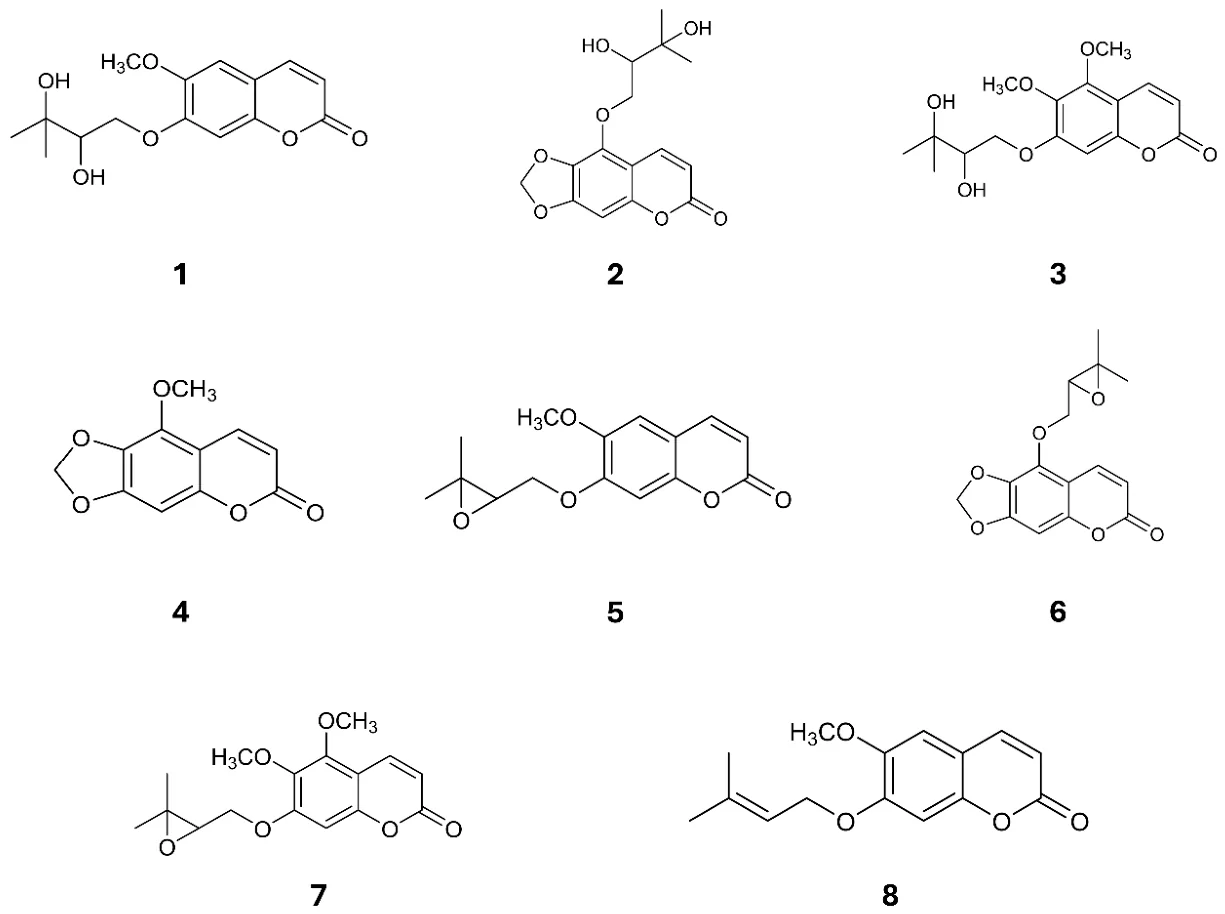
Main compounds identified in the DES extracts from *Pterocaulon polystachyum*, *Pterocaulon balansae*, and *Pterocaulon lorentzii*.

**Figure 8:**
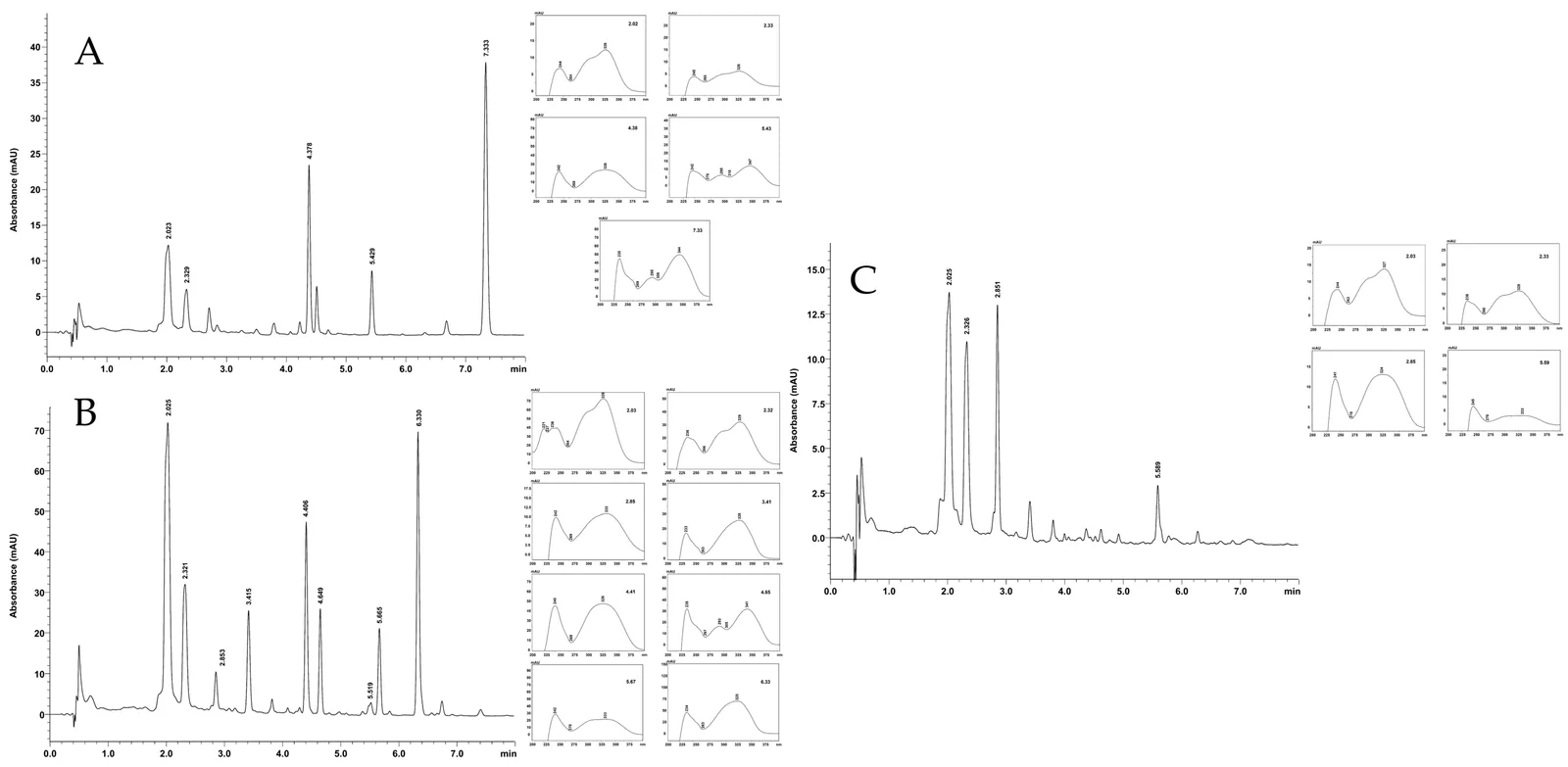
Ultra-Fast Liquid Chromatography (UFLC) chromatogram of the *Pterocaulon polystachyum* (**A**), *P. balansae* (**B**), and *P. lorentzii* (**C**) samples, respectively, showing the chemical profile and the corresponding ultraviolet (UV) absorption spectra of the detected chromatographic peaks.

UFLC-DAD analysis was performed primarily to verify the presence of coumarins (Figure 7) in the DES extracts, with chromatographic peaks tentatively assigned based on their retention times and UV spectra (Figure 8). For *Pterocaulon polystachyum*, two compounds with UV absorption profiles characteristic of phenolic acids were detected at 2.023 and 2.329 min. 5-Methoxy-6,7-methylenedioxycoumarin (**4**) was detected at 4.378 min, closely matching the RT observed for this compound in *P. balansae* DES (4.406 min). In addition, 7-(3-methyl-2-butenyloxy)-6-methoxycoumarin (prenyletin-methyl-ether) (**8**) was detected at 7.333 min. For *P. balansae*, six coumarins were detected and assigned based on their retention times. Sabandinol, 5-(2,3-dihydroxy-3-methylbutyloxy)-6,7-methylenedioxycoumarin (**2**), showed an RT of 2.853 min, whereas 5,6-dimethoxy-7-(3′-methyl-2′,3′-dihydroxybutyloxy)coumarin (**3**) eluted at 3.415 min. The compounds 7-(2′,3′-epoxy-3-methyl-3-butyloxy)-6-methoxycoumarin (**5**), 5-(2′,3′-epoxy-3-methylbutyloxy)-6,7-methylenedioxycoumarin (**6**) and 5,6-dimethoxy-7-(2′,3′-epoxy-3-methylbutyloxy)coumarin (**7**) presented RTs of 4.649, 5.665 and 6.330 min, respectively. Another highlight is that Compound (**1**) (7-(2′,3′-dihydroxy-3-methylbutyloxy)-6-methoxycoumarin) typically elutes at approximately 2.3 min. However, both the peak at 2.321 min and the one at 2.025 min exhibit the UV absorption profile of a phenolic acid. The peak of compound (**1**) is likely overlapped by the peak of the phenolic acid derivative. Finally, sabandinol (**2**) was identified at an RT of 2.851 min in *P. lorentzii*, which was very close to the RT observed for the same compound in *P. balansae* (2.853 min). Compound (**6**), 5-(2′,3′-epoxy-3-methylbutyloxy)-6,7-methylenedioxycoumarin, was detected at 5.589 min, also showing a retention behavior consistent with that observed in *P. balansae* (5.665 min). In addition, peaks with UV absorption profiles characteristic of phenolic acids were also detected in the *P. lorentzii* extract at 2.025 and 2.351 min.

Overall, the close agreement between the retention times observed for the same compounds in different *Pterocaulon* species supports their chromatographic assignment by comparison with literature [7,11,34]. In particular, sabandinol (**2**), coumarin (**6**), and 5-methoxy-6,7-methylenedioxycoumarin (**4**) showed highly similar retention times across the analyzed species, reinforcing their identification based on chromatographic and UV spectral characteristics.

Regarding the proportions of the characterized coumarins, which were estimated from the relative peak areas at 327 nm in the UFLC-DAD chromatograms and, in the absence of calibration curves, should be interpreted as semi-quantitative estimates of the relative abundance of each compound, the following data were obtained: in *P. polystachyum*, compounds **8** and **4** were detected in a 70:30 ratio; in *P. lorentzii*, compounds **2** and **6** were present in an 80:20 ratio; and in *P. balansae*, compounds **7**, **4**, **3**, **5**, **6**, and **2** were characterized in approximate ratios of 36%, 23%, 13%, 12%, 11%, and 4%, respectively.

### 2.3. Mathematical Modeling of the Extractions

**Figure 9:**
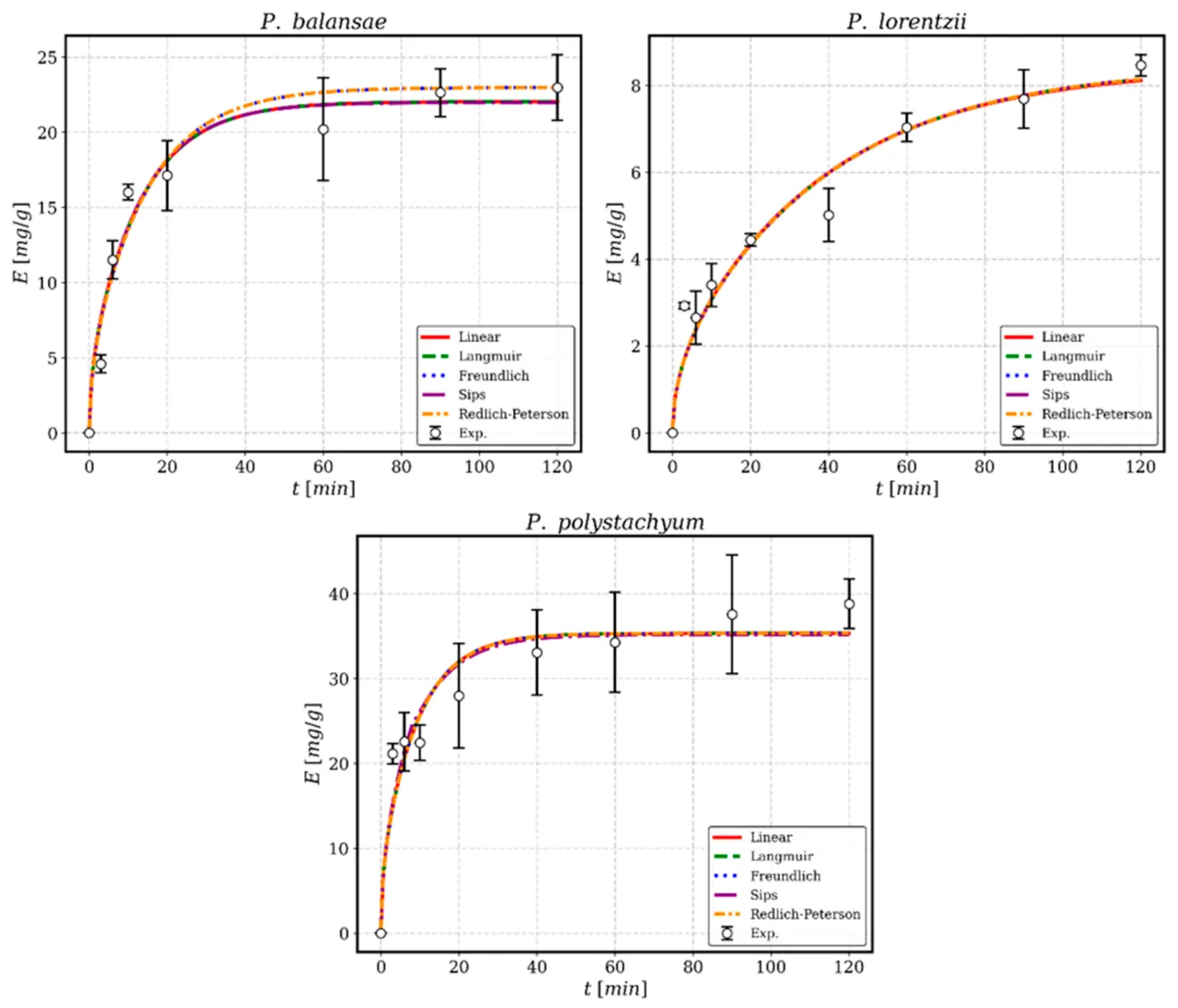
Extraction curves displaying the experimental data and the curves generated with the mathematical model considering different equilibrium isotherms for *P. balansae*, *P. polystachyum*, and *P. lorentzii*.

Extraction curves (Figure 9) are characterized by two distinct stages: a rapid initial phase (washing), lasting up to 10 min, followed by a slower step known as the diffusional stage. The initial rapid extraction occurs at a constant rate, attributed to the immediate exposure of plant cells to the solvent. In contrast, the diffusion stage involves the gradual release of active compounds from the plant matrix into the solvent via molecular transport. Extraction yield during this second phase depends primarily on the number of intact cells remaining after the washing step [26]. Additionally, some studies suggest that the ratio of ruptured to intact cells following sample pretreatment determines the behavior of these two extraction stages [35].

The use of mathematical models that adequately represent the extraction process allows for the evaluation of scale-up and process design without the need for experiments, thereby avoiding resource expenditure. DES extraction was performed in batch mode. Mathematical models for extraction in this mode include models based on sorption principles [36] and mass transfer models [27,30]. In this work, the proposed model for representing DES extraction is based on mass transfer principles and accounts for the variation in the amount of extract in both the fluid and solid phases over time; this resulted in a time-dependent boundary condition, assuming a lumped model for the fluid phase. This approach makes the model effective for representing the physical phenomena occurring during batch extraction with DES.

Other authors have also reported on extraction using deep eutectic solvents and employed models based on extraction stages, including a diffusive step [28,29]. Furthermore, the diffusional model applied in the present work was also used by other authors [24,30] to model intensified extraction techniques from plant material, as previously mentioned.

The analysis of interfacial equilibrium at the solid–liquid interface provides a means of investigating how effective partitioning behavior influences the description of extraction kinetics for different plant matrices. In this context, nonlinear equilibrium relations were evaluated not only as alternative fitting functions but also as a means of testing whether deviations from an approximately constant solid–liquid partition relationship were supported by the available kinetic data. While the linear model assumes direct proportionality between the effective solid- and liquid-phase state variables, nonlinear relations can represent concentration-dependent partitioning, finite solid-phase capacity, and heterogeneous equilibrium behavior [37]. Comparison among these formulations therefore allows the adequacy of a simple partition relationship to be assessed against more flexible nonlinear descriptions.

Among the nonlinear relations evaluated, the Langmuir model introduces a finite maximum capacity, q_m_, whereas the Freundlich equation introduces a concentration-dependent equilibrium relationship. The Sips equation combines features of the Freundlich and Langmuir models by allowing heterogeneous behavior together with a finite limiting capacity. The Redlich–Peterson model provides additional empirical flexibility through its three equilibrium parameters, although the physicochemical interpretation of these parameters may become limited when they are estimated simultaneously from a small kinetic dataset [37,38,39].

Although several nonlinear models provided R^2^ and MAPE values similar to those obtained with the Linear model, model selection changed when parameter complexity was considered through the corrected Akaike Information Criterion (AICc). The Linear model presented the lowest AICc for all three plant matrices. For *P. balansae*, for example, the Linear and Sips models yielded very similar coefficients of determination R^2^ = 0.9593 and 0.9597, respectively, whereas their AICc values were 19.42 and 47.34. A similar trend was observed for *P. lorentzii* and *P. polystachyum*, for which the Linear model also provided the minimum AICc. These results indicate that the additional parameters introduced by the nonlinear equilibrium relations are not sufficiently supported by the available kinetic information to justify their increased complexity. Thus, within the investigated experimental range, the effective solid–liquid partitioning can be described parsimoniously by an approximately linear relationship.

The estimated parameters (Table 4) of the more highly parameterized models also indicate practical-identifiability limitations. For example, some Freundlich, Sips, and Redlich–Peterson parameters converged to extremely small or large numerical values while producing extraction curves very similar to those obtained with simpler formulations. Such behavior suggests that different parameter combinations can compensate for one another during simultaneous estimation and should not be interpreted as direct evidence of specific physicochemical properties of the plant matrix. Accordingly, these coefficients are interpreted primarily as effective model parameters rather than independently identifiable equilibrium properties.

The effective diffusion coefficients, D_eff_, remained on the order of 10^−11^ m^2^/s for all plant matrices. Considering all equilibrium formulations, the estimated values ranged from approximately 2.06 × 10^−11^ m^2^/s for *P. polystachyum* to 7.85 × 10^−11^ m^2^/s for *P. balansae*. However, D_eff_ was estimated simultaneously with the equilibrium parameters and should therefore be interpreted as a model-dependent effective transport coefficient rather than as an intrinsic molecular diffusivity. The dependence of its fitted value on the selected equilibrium relation reflects the coupling between intraparticle transport and interfacial partitioning in the inverse estimation procedure.

Overall, the experimental extraction curves were adequately represented by the effective diffusion-based formulation. Nevertheless, agreement between the model and the experimental data does not independently demonstrate that intracellular or intraparticle diffusion is the unique rate-controlling mechanism. Rather, the results indicate that the observed extraction kinetics are compatible with an effective Fickian description under the investigated conditions, with the simpler Linear equilibrium relation providing the most parsimonious representation of the available data.

## 3. Materials and Methods

### 3.1. Preparation of DESs

The DES used in this study was betaine:ethylene glycol 1:3, since it was previously reported for coumarin extraction from *Pterocaulon* species [13]. The moisture content of this DES was reported in our previous work as 0.65% [13] under the same reagents and preparation techniques. This particular DES was also compared with other solvents and tested for toxicity against HaCaT cells in our previous study, showing promising and safe results for potential application in the pharmaceutical and cosmetic industries due to the presence of phenolic compounds and coumarins in the extract [13].

It was prepared according to the heating and stirring method [40]. Its constituent compounds (HBA and HBD—Table 5) were weighed on an analytical balance according to predetermined molar ratios and mixed at a controlled temperature (50 °C) for 2.5 h. The formation of a clear, homogeneous solution with no visible crystal formation was observed. Prior to the extraction experiments, the DES was stored at room temperature for 24 h to ensure stabilization.

### 3.2. Plant Material

The aerial parts of *P. polystachyum* and *P. balansae* used in this study were collected in the municipality of Nova Santa Rita, located in southern Brazil (29°51′25″ S, 51°16′26″ W), while those of *P. lorentzii* were collected in the municipality of Xangri-lá (29°48′03″ S, 50°02′38″ W), also located in southern Brazil. Plants were collected in February 2025 and identified by Dr. Sérgio A. L. Bordignon (Centro Universitário La Salle-Canoas, Unilasalle, Brazil). Vouchers were deposited in the herbarium of the Universidade Federal do Rio Grande do Sul (ICN) (*Pterocaulon balansae*: ICN 157762; *P. lorentzii*: ICN 140005; *P. polystachyum*: ICN 136584). The plant collection was registered in the Sistema Nacional de Gestão do Patrimônio Genético (SISGEN). The plant material was oven-dried at 40 °C for 24 h until reaching a constant mass. The final water content was determined using a Bel Engineering thermogravimetric moisture analyzer (model i-Thermo 163 L), Monza, Italy. Then the plant material was milled in a knife grinder. The mean particle diameter was evaluated using five Tyler series sieves (Endecotts BS410:1986 Test Sieve) with 10 min of agitation. The target granulometry was achieved by mixing selected fractions from different particle size ranges. The plant density was determined via gas pycnometry technique (Quantachrome Multipycnometer), Boynton Beach, FL, USA [41]. Data are summarized in Table 6.

### 3.3. Experimental Design

The planning and optimization of ultrasound-assisted extraction parameters using deep eutectic solvents (DES) were carried out using response surface methodology via a Box–Behnken design in Minitab^®^ software (Table 6). For the different *Pterocaulon* species, previously optimized parameters such as the solid-to-liquid (S/L) ratio in g/mL and temperature were fixed based on recent literature [13] (0.05 and 40 °C), allowing the focus to shift to the optimization of other factors. Each extraction was performed using 2 mL of DES and 100 mg of ground plant material. Although these optimal points were determined only for *P. polystachyum*, they were also extrapolated to *P. balansae* and *P. lorentzii* in this study, ensuring uniformity in the variables examined across the different plant matrices and enabling the investigation of other variables related to plant pre-treatment.

The significance of the model, the parameters, and their interactions within the extraction process were evaluated using analysis of variance (ANOVA). The three independent factors evaluated and their respective levels were:

- Extraction time: 30, 60, and 90 min;
- Pretreatment ultrasonic power (probe pretreatment power only): 165 W, 220 W, and 275 W (corresponding to 30%, 40%, and 50% of the 550 W equipment’s maximum power, respectively);
- Pretreatment cycles: 4, 8, and 12 ultrasonic cycles (with a cycle defined as 10 s on and 60 s off).

Each standard order represents one single extraction. During pretreatment, temperature amplitude and energy delivered were not recorded. Additionally, we highlight that, from this point on, a pretreatment ultrasonic power optimization variable may sometimes be referred to simply as “power”.

### 3.4. Application of Ultrasonication Pretreatment

Prior to the actual extraction, the plant material samples—already mixed with the deep eutectic solvent (DES)—underwent ultrasonic pretreatment. A high-intensity probe sonicator (Ultronique, QR550, 20 kHz—5 mm diameter tip) operating at a maximum power of 550 W was used. The pretreatment involved applying ultrasonic pulses directly to the solid–liquid mixture, submerging the tip in the liquid, avoiding contact with the tube walls and bottom of the 5 mL microtube, cycles of 10 s on and 60 s off, with power levels ranging from 165 W to 275 W, in accordance with the experimental design specific to each species of the genus *Pterocaulon*. The microtubes were kept in a beaker with water and ice during the application of the pretreatment.

### 3.5. Ultrasound-Assisted Extraction

Ultrasound-assisted extraction (UAE) coupled with DES was used to obtain extracts from all the plants, as this approach has previously been reported to enhance extraction efficiency and reduce processing time compared to other methods [42]. UAE was performed using a digital ultrasonic bath with temperature control (Ultronique, model Q 5.9/40 A, 40 kHz, 200 W, standard uncertainty u(T) = 3.00 K) Indaiatuba, Brazil, a methodology recommended for a wide variety of samples under controlled temperature conditions [40]. It is important to highlight that the ultrasonic power during extraction was not evaluated in the Box–Behnken design. The bath water volume was 1625 cm^3^. The samples were placed in 5 mL Eppendorf tubes with a center-to-center distance of 3 cm. Extraction parameters were determined using a Box–Behnken experimental design in single experiments. Extraction curves were generated (triplicates); following extraction, the samples were filtered through cotton in a 3 mL syringe, and the resulting extracts were analyzed using liquid chromatography and spectrophotometric methods.

### 3.6. Analysis of the Extracts

#### 3.6.1. Total Phenolic Content (TPC)

Total phenolic content (TPC) of the extracts with DES was determined by the Folin–Ciocalteu colorimetric method [43,44,45]. The methodology consists of adding 20 μL of the samples (DES extracts), 1.58 mL of deionized water, 100 μL of Folin–Ciocalteu reagent, and 300 μL of an aqueous sodium carbonate solution (0.2 g/mL) to glass test tubes. The mixture was then vortexed and incubated in a water bath for 30 min at 40 °C. Sample absorbance was measured using UV-Vis spectrophotometry (wavelength of 750 nm), and a calibration curve was constructed using gallic acid standard solution concentrations of 0.025, 0.05, 0.10, 0.25, 0.50, and 1.0 g/L. The blank was prepared using only the DES as the sample to ensure that the solvent does not interfere with the measurement. Results for total phenolic content were expressed as milligrams of gallic acid equivalents (mg GAE) per gram of plant weight.

#### 3.6.2. Ultra-Fast Liquid Chromatography (UFLC) Analysis

Coumarin analysis was performed by UFLC-DAD using a previously validated method [11], with minor adaptations as required for the present samples. Briefly, analyses were conducted using a Shimadzu Prominence UFLC system (Shimadzu, Japan) equipped with a diode array detector (SPD-M20A) set at 327 nm. The chromatographic data were acquired and processed using Shimadzu LC-solution Multi-PDA software (Kyoto, Japan). Separation was achieved on a Shim-pack XR C18 column (100 × 2.0 mm, 2.2 μm) protected by a C18 SecurityGuard ULTRA pre-column (Phenomenex, Torrance, CA, USA). The mobile phase consisted of 0.1% formic acid (*v*/*v*) (A) and acetonitrile (B), delivered according to the following gradient: 0–17% B (0–0.09 min), 17–25% B (0.09–1.45 min), 25% B (1.45–2.20 min), 25–32% B (2.20–2.90 min), 32–37% B (2.90–4.77 min), 37–39% B (4.77–4.90 min), 39–40% B (4.90–5.60 min), 40% B (5.60–7.20 min), 40–22% B (7.20–7.80 min), and 22–17% B (7.80–8.00 min). The column was subsequently re-equilibrated at 17% B for 2 min. The flow rate was 0.55 mL/min, the column temperature was maintained at 55 °C, and the injection volume was 5 μL.

Coumarins were assigned based on their chromatographic retention times and UV absorption spectra, according to the previously established method [11]. The analytical procedure had previously been demonstrated to be selective, linear, precise, accurate, and robust [11].

### 3.7. Mathematical Modeling

The batch extraction process was described using an effective one-dimensional intraparticle diffusion model based on Fick’s second law. The ground plant particles were represented by an equivalent slab of half-thickness (L), defined as one-half of the experimentally determined mean particle diameter.

This geometrical assumption does not imply that the ground plant particles have an actual planar shape. Rather, the slab geometry provides an idealized characteristic diffusion path for the heterogeneous plant matrix, with unresolved morphological and structural effects incorporated into the effective transport parameter. The liquid phase was assumed to be perfectly mixed, resulting in a spatially uniform liquid-phase state variable coupled to the particle surface through a time-dependent boundary condition [24].

Because total phenolic content (TPC) represents an aggregate response of a chemically heterogeneous extract, the model variables q and c are interpreted as effective TPC-equivalent state variables associated with the solid and liquid phases, respectively, rather than as concentrations of a single chemical compound. The effective diffusion coefficient, D_eff_, was assumed to remain constant over the extraction interval investigated. Accordingly, D_eff_ should be interpreted as a lumped parameter that incorporates unresolved effects associated with the heterogeneous plant matrix, including solvent penetration, matrix swelling, and the simultaneous transport of the compounds contributing to the measured TPC response.

Under these assumptions, the governing equation with the dynamic boundary condition at the interface is:

(1) $$\frac{\partial q}{\partial t}=D_{eff}\frac{\partial^{2}q}{\partial x^{2}}$$

(2) $$q(x,0)=q_{0}$$

(3) $${\left.\frac{\partial q}{\partial x}\right|}_{x=0}=0\;\;\;\;\;{\left.-D_{eff}\frac{\partial q}{\partial x}\right|}_{x=L}=\frac{V_{L}}{V_{s}a_{p}}\frac{\partial c}{\partial t}$$

where *q* and *c* are effective TPC-equivalent state variables associated with the solid and liquid phases, respectively, expressed on a dry-plant-mass basis (mg GAE/g dry plant). *D_eff_* is the effective diffusion coefficient (m^2^/s), $V_{L}$ is the solvent volume used in the extraction (m^3^), $V_{s}$ is the solid particles volume (m^3^), calculated from the mass of particles (*W*—[kg]) used with Vs=Wρs in which $\rho_{s}$ is the solid density (kg/m^3^) and $a_{p}$ is the specific surface (m^−1^) which relates the surface area with the volume of the particle as $a_{p}=1/L$.

The initial solid-phase extractable content, *q*_0_, was defined from the experimentally determined asymptotic extraction yield obtained under exhaustive extraction conditions (considered when the extraction curves appeared to approach a plateau). The values adopted were 38.79, 22.97, and 8.46 mg GAE/g of plant material for *P. polystachyum*, *P. balansae*, and *P. lorentzii*, respectively. These values represent the total extractable content initially available in the solid matrix within the TPC-equivalent representation of the model.

The solid and liquid phases concentration at the interface of the particle are treated with different equilibrium relations based on isotherms, with a general form given by:

(4) $$q=f\left(c\right)$$

The different equilibrium isotherms used in the present work are presented in Table 7.

Where q is the concentration of solute in the solid phase and c is the concentration in the liquid phase. For the Linear model, K represents the equilibrium constant. In the Langmuir model, $q_{m}$ denotes the maximum adsorption capacity, and $K_{L}$ is the adsorption constant related to the affinity of the binding sites. For the Freundlich equation, k is the constant related to adsorption capacity, and n is the heterogeneity factor. Regarding the Sips model, $q_{m}$ is the maximum capacity, $K_{s}$ is the Sips equilibrium constant, and α is the heterogeneity index. Finally, in the Redlich-Peterson model, $K_{rp}$ is the model constant, β is the affinity coefficient, and γ is the exponent that characterizes the system between the Langmuir and Freundlich limits [37]. Parameter units follow directly from the dimensional form of each equilibrium relation. Considering *q* and *c* expressed on the same TPC-equivalent basis, K and $K_{rp}$ are dimensionless, $q_{m}$ has the same units as q, $K_{L}$ has units of c^−1^, k has units of [q][c]^−1/n^, $K_{s}$ has units of [c]^−1/α^, and β has units of [c]^−γ^. The exponents n, α, and γ are dimensionless.

Since the inclusion of non-linear equilibrium isotherms prevents a straightforward analytical solution, a numerical approach was implemented to solve the coupled system of equations. A numerical method was employed to solve the coupled mass-transfer model for all equilibrium relations. The model was implemented in Python using NumPy and SciPy. The spatial domain was discretized by finite differences, with second-order central differences applied to the internal nodes [46]. The symmetry condition at x = 0 was incorporated directly into the corresponding finite-difference boundary expression. At the solid–liquid interface, the diffusive flux evaluated at the external node was directly coupled to the well-mixed liquid-phase balance. The dynamic equilibrium condition was imposed through the derivative of the selected equilibrium relation (dq/dc).

Spatial discretization resulted in a system of ordinary differential equations, which was integrated in time using the backward differentiation formula (BDF). Relative and absolute integration tolerances of 10^−8^ and 10^−10^, respectively, were adopted. A 31-node spatial discretization was used during parameter estimation and calculation of statistical metrics, whereas 51 nodes were used to generate the final smooth model curves.

The effective diffusion coefficient and the parameters associated with each equilibrium relation were estimated simultaneously by minimizing the unweighted mean squared error between the experimental extraction yields and the corresponding model predictions:

(5) $$MSE\left(\theta \right)=\frac{1}{N}\sum_{i=1}^{N}{\left[E_{mod}\left(t_{i};\theta \right)-E_{exp,i}\right]}^{2}$$

where *N* is the number of experimental observations and θ is the vector containing *D_eff_* and the parameters of the selected equilibrium relation. Experimental standard deviations were used to represent the uncertainty of the experimental points graphically but were not used as weights in the objective function. Parameter estimation was performed using the Nelder–Mead algorithm implemented in Python (SciPy), with a convergence tolerance of 10^−9^.

Because the equilibrium models contain different numbers of adjustable parameters, model comparison based exclusively on R^2^ and MAPE may favor more highly parameterized formulations. Therefore, model comparison was complemented using the corrected Akaike Information Criterion, which accounts for model complexity and includes a finite-sample correction suitable when the number of observations is small relative to the number of estimated parameters.

For least-squares fitting, *AIC* was calculated as:

(6) $$AIC=N\ln\left(\frac{RSS}{N}\right)+2\eta$$

and its small-sample corrected form as:

(7) $$AIC_{c}=AIC+\frac{2\eta \left(\eta +1\right)}{N-\eta -1}$$

where *RSS* is the residual sum of squares and *η* is the total number of estimated parameters in the statistical model, including the residual variance.

### 3.8. Statistical Analysis

For the extracts obtained at 90 and 120 min in the extraction curves for all species, analyses were performed using the Minitab 19 software by one-way analysis of variance (ANOVA), followed by a Tukey test, where mean values of the triplicates were considered significantly different at *p* < 0.05 (95% confidence interval).

## 4. Conclusions

Based on the results obtained, we can conclude that ultrasound-assisted extraction using deep eutectic solvents (DES) proved to be an effective strategy for recovering phenolic compounds from the species *Pterocaulon polystachyum*, *P. balansae,* and *P. lorentzii*, as well as coumarins, which were tentatively identified by UFLC-DAD based on their retention times and UV absorption spectra. Optimization via Box–Behnken design enabled the identification of specific conditions that maximize TPC for each plant matrix within the investigated range, highlighting the influence of each species’ structural and chemical characteristics on the process. Mathematical modeling based on effective diffusion adequately represented the extraction kinetics, indicating that the observed behavior is compatible with an effective Fickian description under the investigated conditions; however, this agreement alone does not demonstrate that diffusion is the sole rate-controlling step. Because all experiments were performed at laboratory scale (100 mg of plant material and 2 mL of DES), the estimated parameters should be regarded as a preliminary basis for future scale-up studies rather than as directly transferable design values. Under the optimized conditions, the maximum TPC values reached 37.56 ± 7.01, 22.63 ± 1.60, and 7.69 ± 0.67 mg GAE/g for *P. polystachyum*, *P. balansae*, and *P. lorentzii*, respectively, compared with 32.01 ± 0.01, 6.75 ± 2.82, and 5.44 ± 0.93 mg GAE/g obtained without probe pretreatment. These results underscore the potential of this system for pharmaceutical and cosmetic applications, provided that complementary analyses and tests specific to this purpose are conducted, aligning with green chemistry principles and the demand for selective, low-environmental impact processes.

## Figures and Tables

**Table 1:** Three-Factor Box–Behnken Design for *Pterocaulon* sp. with TPC results.

Order	Extraction Time (min)	Power (W)	Nº of Cycles	TPC * P. polystachyum	TPC * P. balansae	TPC * P. lorentzii
1	30	165	8	20.80	9.47	1.62
2	30	275	8	23.36	10.65	5.39
3	30	220	4	20.91	8.78	3.03
4	30	220	12	19.31	7.98	4.70
5	60	165	4	18.64	16.16	3.78
6	60	275	4	32.24	24.53	6.02
7	60	165	12	23.78	14.89	2.95
8	60	275	12	32.83	17.64	3.92
9	60	220	8	20.86	16.17	3.03
10	60	220	8	26.73	12.55	4.07
11	60	220	8	21.17	6.48	3.34
12	90	165	8	34.51	17.98	3.12
13	90	275	8	34.66	19.65	3.78
14	90	220	4	24.80	14.09	2.95
15	90	220	12	24.21	7.02	3.93

* TPC expressed in mg GAE per gram of plant.

**Table 2:** Regression models and fitting parameters for total phenolic content (TPC) in different species.

Species	Regression Equation (Uncoded Units)	R^2^ (%)	R^2^_adj_ (%)	R^2^_pred_ (%)	RMSE (mg GAE/g)	CV (%)	F_model_	p_model_	Lack-of-Fit *p*-Value
*P. polystachyum*	TPC = 65.3 + 0.148TE − 0.604PT + 2.15NC + 0.00047TE^2^ + 0.001648PT^2^ − 0.065NC^2^ − 0.00037TE*PT + 0.0021TE*NC − 0.0052PT*NC	74.67	29.07	0.00	2.74	18.77	1.64	0.305	0.277
*P. balansae*	TPC = 63.2 − 0.190TE − 0.379PT − 2.33NC + 0.00036TE^2^ + 0.000428PT^2^ − 0.099NC^2^ + 0.00109TE*PT + 0.0074TE*NC + 0.0172PT*NC	70.89	18.49	0.00	2.72	34.69	1.35	0.387	0.852
*P. lorentzii*	TPC = 17.55 − 0.1347TE − 0.0701PT − 0.924NC + 0.000022TE^2^ + 0.000072PT^2^ − 0.0176NC^2^ + 0.000364TE*PT + 0.00990TE*NC + 0.002908PT*NC	96.09	89.06	60.38	0.206	9.62	13.66	0.005	0.565

**Table 3:** Summary table presenting moisture content, particle diameter (PD) in mm, and plant density.

Species	% Moisture	PD (mm)	ρ (g/cm^3^)
*P. polystachyum*	4.89 ± 1.87	0.352	1.240 ± 0.024
*P. balansae*	5.76 ± 0.15	0.779	1.152 ± 0.027
*P. lorentzii*	7.17 ± 1.15	0.871	1.089 ± 0.054

**Table 4:** Estimated parameters for DES extraction of different plant materials and statistical metrics assessing the quality of fit *.

Plant Material	Model	R^2^	MAPE	AICc	D × 10^−11^	K	K_L_	k	n	q_m_	K_s_	α	K_rp_	β	γ
			[%]		[m^2^/s]										
*P. balansae*	Linear	0.9593	15.4	19.42	7.44	1.00	-	-	-	-	-	-	-	-	-
	Langmuir	0.9593	15.4	28.75	7.44	-	0.0183	-	-	55.4	-	-	-	-	-
	Freundlich	0.9544	15.2	29.66	6.96	-	-	2.18 × 10^−10^	7.20 × 10^−12^	-	-	-	-	-	-
	Sips	0.9597	15.4	47.34	7.85	-	-	-	-	3.07 × 10^9^	0.272	1.49 × 10^10^	-	-	-
	Redlich-Peterson	0.9544	15.2	48.32	6.96	-	-	-	-	-	-	-	5.77 × 10^−4^	1.53 × 10^−10^	1.18 × 10^−7^
*P. lorentzii*	Linear	0.9529	11.2	0.334	3.29	9.56	-	-	-	-	-	-	-	-	-
	Langmuir	0.9529	11.3	7.538	3.25	-	2.50 × 10^−11^	-	-	1.45 × 10^−10^	-	-	-	-	-
	Freundlich	0.9529	11.3	7.537	3.26	-	-	2.96 × 10^−5^	7.40 × 10^−4^	-	-	-	-	-	-
	Sips	0.9529	11.3	19.54	3.26	-	-	-	-	0.359	1.66 × 10^−8^	26.2	-	-	-
	Redlich-Peterson	0.9529	11.3	19.54	3.26	-	-	-	-	-	-	-	3.18 × 10^−4^	1.18 × 10^−10^	6.79 × 10^−5^
*P. polystachyum*	Linear	0.9178	11.5	31.90	2.06	0.405	-	-	-	-	-	-	-	-	-
	Langmuir	0.9182	11.4	39.06	2.11	-	0.475	-	-	8.66	-	-	-	-	-
	Freundlich	0.9178	11.5	39.10	2.06	-	-	2.49	1.03	-	-	-	-	-	-
	Sips	0.9229	10.8	50.52	2.06	-	-	-	-	3.63	1.80	1.24 × 10^−4^	-	-	-
	Redlich-Peterson	0.9182	11.4	51.06	2.11	-	-	-	-	-	-	-	4.11	0.475	1.00

* Parameter units follow directly from the dimensional form of each equilibrium relation. Considering *q* and *c* expressed on the same TPC-equivalent basis, K and $K_{rp}$ are dimensionless, $q_{m}$ has the same units as q, $K_{L}$ has units of c^−1^, k has units of [q][c]^−1/n^, $K_{s}$ has units of [c]^−1/α^, and β has units of [c]^−γ^. The exponents n, α, and γ are dimensionless.

**Table 5:** CAS number, source, supplier purity, and molar mass of the compounds used to form the DES.

Compound	CAS Number	Source	Purity *^a^*	Molar Mass (g/mol)
Betaine	107-43-7	Sigma-Aldrich (St. Louis, MO, USA)	≥99.0%	117.15
Ethylene glycol	107-21-1	Sigma-Aldrich (St. Louis, MO, USA)	≥99.0%	62.07

*^a^* Purity as reported by the supplier. The reagents were used without further purification.

**Table 6:** Three-Factor Box–Behnken Design for *Pterocaulon* sp.

Standard Order	Extraction Time (min)	Pretreatment Ultrasonic Power (W)	Pretreatment Cycles
1	30	165	8
2	30	275	8
3	30	220	4
4	30	220	12
5	60	165	4
6	60	275	4
7	60	165	12
8	60	275	12
9	60	220	8
10	60	220	8
11	60	220	8
12	90	165	8
13	90	275	8
14	90	220	4
15	90	220	12

**Table 7:** Equilibrium isotherms used to represent the partition of solute between the liquid interface and the solid particle.

Isotherm	Equilibrium Model $q=f\left(c\right)$
Linear	q=c/K
Langmuir	$q=q_{m}\frac{K_{L}c}{1+K_{L}c}$
Freundlich	$q=kc^{1/n}$
Sips	$q=q_{m}\frac{K_{s}c^{1/\alpha }}{1+K_{s}c^{1/\alpha }}$
Redlich—Peterson	$q=\frac{K_{rp}c}{1+\beta c^{\gamma }}$

## Data Availability

All the available data are reported in this work.

## Supplementary Materials

The following supporting information can be downloaded at: https://www.mdpi.com/article/10.3390/molecules31213849/s1.
