# Supplementary material for: Optimization and Mathematical Modeling of Biocompound Extraction from Pterocaulon Species Using Deep Eutectic Solvents
Source: Molecules. 2026 Oct 25;31(21):3849. doi: 10.3390/molecules31213849 (PMC13764989; doi:10.3390/molecules31213849)
Supplement: Supplementary file 1 [file molecules-31-03849-s001.zip › molecules-4603666-supplementary.pdf]

ANOVA Table for *Pterocaulon* sp.

POLYSTACHYUM

Response Surface Regression: TPC (mg GAE/ g of planta) versus Extraction time; Power (W); Pretreatment time

Coded Coefficients

Term	Coef	SE Coef	T-Value	P-Value	VIF
Constant	22.92	2.74	8.38	0.000	
Extraction time	4.23	1.68	2.52	0.053	1.00
Power (W)	3.17	1.68	1.89	0.117	1.00
Pretreatment time	0.44	1.68	0.26	0.803	1.00
Extraction time*Extraction time	0.43	2.47	0.17	0.870	1.01
Power (W)*Power (W)	4.99	2.47	2.02	0.099	1.01
Pretreatment time*Pretreatment time	-1.03	2.47	-0.42	0.692	1.01
Extraction time*Power (W)	-0.60	2.37	-0.26	0.809	1.00
Extraction time*Pretreatment time	0.25	2.37	0.11	0.919	1.00
Power (W)*Pretreatment time	-1.14	2.37	-0.48	0.652	1.00

Supplementary Table S1. Coded coefficients of the response surface regression model for total phenolic content (TPC, mg GAE/g of plant) of *P. polystachyum* as a function of extraction time, power (W), and pretreatment time.

Model Summary

S	R2	R2(aj)	R2(pred)
4.73908	74.67%	29.07%	0.00%

Supplementary Table S2. Model summary for the response surface regression of TPC (mg GAE/g of plant) of *P. polystachyum*, including S, R^2, adjusted R^2, and predicted R^2.

Analysis of Variance

Source	DF	Adj SS	Adj MS	F-Value	P-Value
Model	9	331.019	36.780	1.64	0.305
Linear	3	224.792	74.931	3.34	0.114
Extraction time	1	142.900	142.900	6.36	0.053
Power (W)	1	80.333	80.333	3.58	0.117
Pretreatment time	1	1.560	1.560	0.07	0.803
Square	3	99.347	33.116	1.47	0.328
Extraction time*Extraction time	1	0.670	0.670	0.03	0.870
Power (W)*Power (W)	1	91.801	91.801	4.09	0.099
Pretreatment time*Pretreatment time	1	3.952	3.952	0.18	0.692
2-Way Interaction	3	6.880	2.293	0.10	0.955
Extraction time*Power (W)	1	1.463	1.463	0.07	0.809
Extraction time*Pretreatment time	1	0.256	0.256	0.01	0.919
Power (W)*Pretreatment time	1	5.161	5.161	0.23	0.652
Error	5	112.294	22.459		
Lack-of-Fit	3	90.482	30.161	2.77	0.277
Pure Errorr	2	21.812	10.906	*	*
Total	14	443.314			

Supplementary Table S3. Analysis of variance (ANOVA) for the response surface regression model of TPC (mg GAE/g of plant) of *P. polystachyum*, including linear, quadratic, and two-way interaction terms, lack-of-fit, and pure error.

Regression Equation in Uncoded Units

TPC (mg GAE/g of plant) = 65.3 + 0.148 Extraction Time - 0.604 Power (W)
+ 2.15 Pretreatment time
+ 0.00047 Time of Extraction*Time of Extraction
+ 0.001648 Power (W)*Power (W)
- 0.065 Pretreatment time * Pretreatment time
- 0.00037 Extraction Time*Power (W)
+ 0.0021 Extraction Time * Pretreatment time
- 0.0052 Power(W)* Pretreatment time

Supplementary Equation S1. Regression equation in uncoded units for TPC (mg GAE/g of plant) of *P. polystachyum* as a function of extraction time, power (W), and pretreatment time.

POLYSTACHYUM

Response Optimization: TPC (mg GAE/ g of planta)

Parameters

Response	Goal	Lower	Target	Upper	Weight	Importance
TPC (mg GAE/ g of plant)	Maximum	18.6398	34.6564		1	1

Supplementary Table S4. Response optimization parameters for maximizing TPC (mg GAE/g of plant) of *P. polystachyum*.

Solution

Solution	Extraction time	Power (W)	Pretreatment time	TPC (mg GAE/ g of plant) Fit	Desirability Composite
1	90	275	7.15152	35.1682	1

Supplementary Table S5. Response optimization solution for *P. polystachyum*, showing the selected extraction time, power, pretreatment time, fitted TPC, and composite desirability.

Multiple Response Prediction

Variable	Setting
Extraction time	90
Power (W)	275
Pretreatment time	7.15152

**Response**	**Fit**	**SE of the Ajusted**	**95% CI**	**95% PI**
TPC (mg GAE/ g of planta)	35.17	4.15	(24.50; 45.84)	(18.97; 51.36)

Supplementary Table S6. Multiple response prediction for TPC (mg GAE/g of plant) of *P. polystachyum* at the selected extraction time, power, and pretreatment time, including fitted value, standard error of fit, 95% confidence interval, and 95% prediction interval.

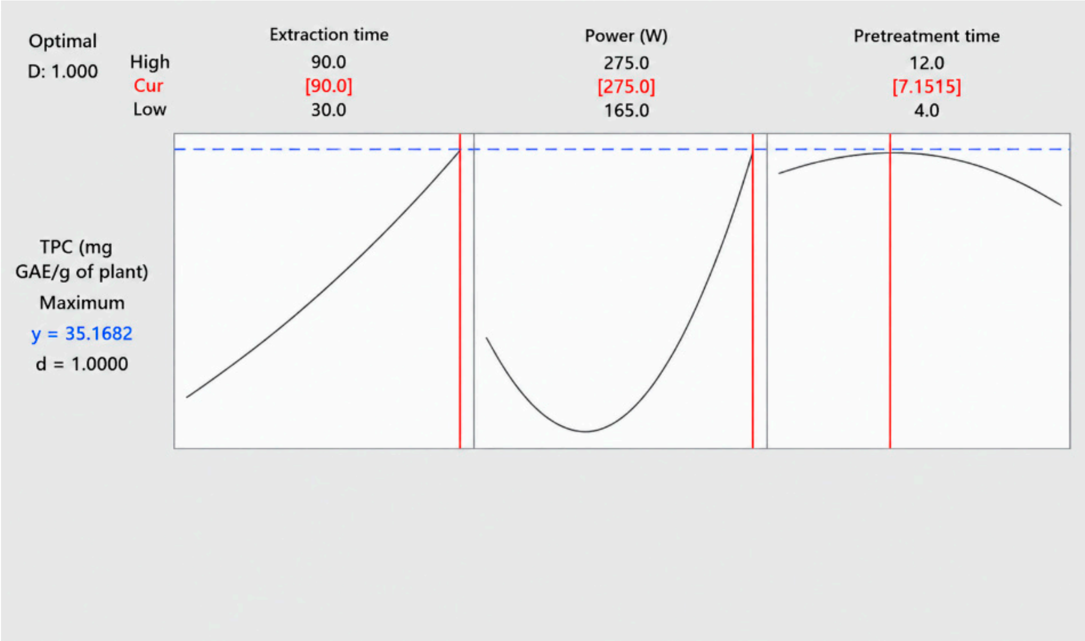

Supplementary Figure S1. Response optimizer plot for TPC (mg GAE/g of plant) of *P. polystachyum*, showing the effects of extraction time, power (W), and pretreatment time and the selected optimal settings (red vertical lines) with composite desirability D = 1.0000.

BALANSAE

Response Surface Regression: TPC (mg GAE/ g of planta) versus Extraction time; Power (W); Pretreatment time

Coded Coefficients

**Term**	**Coef**	**SE Coef**	**T-Value**	**P-Value**	**VIF**
Constant	13.59	2.72	4.99	0.004	
Extraction time	4.54	1.67	2.72	0.042	1.00
Power (W)	0.66	1.67	0.40	0.707	1.00
Pretreatment time	1.23	1.67	0.74	0.494	1.00
Extraction time*Extraction time	0.32	2.46	0.13	0.901	1.01
Power (W)*Power (W)	1.29	2.46	0.53	0.621	1.01

Pretreatment time*Pretreatment time	-1.59	2.46	-0.65	0.546	1.01
Extraction time*Power (W)	1.80	2.36	0.76	0.481	1.00
Extraction time*Pretreatment time	0.89	2.36	0.38	0.722	1.00
Power (W)*Pretreatment time	3.78	2.36	1.60	0.170	1.00

Supplementary Table S7. Coded coefficients of the response surface regression model for TPC (mg GAE/g of plant) of *Pterocaulon balansae* as a function of extraction time, power (W), and pretreatment time.

Model Summary

S	R2	R2(aj)	R2(pred)
4.71870	70.89%	18.49%	0.00%

Supplementary Table S8. Model summary for the response surface regression of TPC (mg GAE/g of plant) of *P. balansae*, including S, R^2, adjusted R^2, and predicted R^2.

Analysis of Variance

Source	DF	Adj SS	Adj MS	F-Value	P-Value
Model	9	271.115	30.124	1.35	0.387
Linear	3	180.668	60.223	2.70	0.156
Extraction time	1	165.070	165.070	7.41	0.042
Power (W)	1	3.519	3.519	0.16	0.707
Pretreatment time	1	12.079	12.079	0.54	0.494
Square	3	17.193	5.731	0.26	0.853
Extraction time*Extraction time	1	0.378	0.378	0.02	0.901
Power (W)*Power (W)	1	6.179	6.179	0.28	0.621
Pretreatment time*Pretreatment time	1	9.314	9.314	0.42	0.546
2-Way Interaction	3	73.254	24.418	1.10	0.432
Extraction time*Power (W)	1	12.917	12.917	0.58	0.481
Extraction time*Pretreatment time	1	3.153	3.153	0.14	0.722
Power (W)*Pretreatment time	1	57.184	57.184	2.57	0.170
Error	5	111.331	22.266		
Lack-of-Fit	3	31.142	10.381	0.26	0.852
Pure Errorr	2	80.189	40.095	*	*
Total	14	382.446			

Supplementary Table S9. Analysis of variance (ANOVA) for the response surface regression model of TPC (mg GAE/g of plant) of *P. balansae*, including linear, quadratic, and two-way interaction terms, lack-of-fit, and pure error.

Regression Equation in Uncoded Units

TPC (mg GAE/ g of planta)	=	63.2 - 0.190 Extraction Time - 0.379 Power (W)<br>- 2.33 Pretreatment time<br>+ 0.00036 Extraction time * Extraction time<br>+ 0.000428 Power (W)* Power (W)<br>- 0.099 Pretreatment time * Pretreatment time<br>+ 0.00109 Extraction time * Power (W)<br>+ 0.0074 Extraction time * Pretreatment time<br>+ 0.0172 Power (W)* Pretreatment time

Supplementary Equation S2. Regression equation in uncoded units for TPC (mg GAE/g of plant) of *P. balansae* as a function of extraction time, power (W), and pretreatment time.

BALANSAE

Response Optimization: TPC (mg GAE/ g of planta)

Parameters

Response	Goal	Lower	Target	Upper	Weight	Importance
TPC (mg GAE/ g of plant)	Maximum	6.47834	24.5318		1	1

Supplementary Table S10. Response optimization parameters for maximizing TPC (mg GAE/g of plant) of *P. balansae*.

Solution

Solution	Extraction time	Power (W)	Pretreatment time	TPC (mg GAE/ g of plant) Fit	Desirability Composite
1	90	275	12	26.5144	1

Supplementary Table S11. Response optimization solution for *P. balansae*, showing the selected extraction time, power, pretreatment time, fitted TPC, and composite desirability.

Multiple Response Prediction

Variable	Setting
Extraction time	90
Power (W)	275
Pretreatment time	12

Response	Fit	EP do Ajustado	95% CI	95% PI
TPC (mg GAE/ g of planta)	26.51	5.57	(12.18; 40.85)	(7.74; 45.29)

Supplementary Table S12. Multiple response prediction for TPC (mg GAE/g of plant) of *P. balansae* at the selected extraction time, power, and pretreatment time, including fitted value, standard error of fit, 95% confidence interval, and 95% prediction interval.

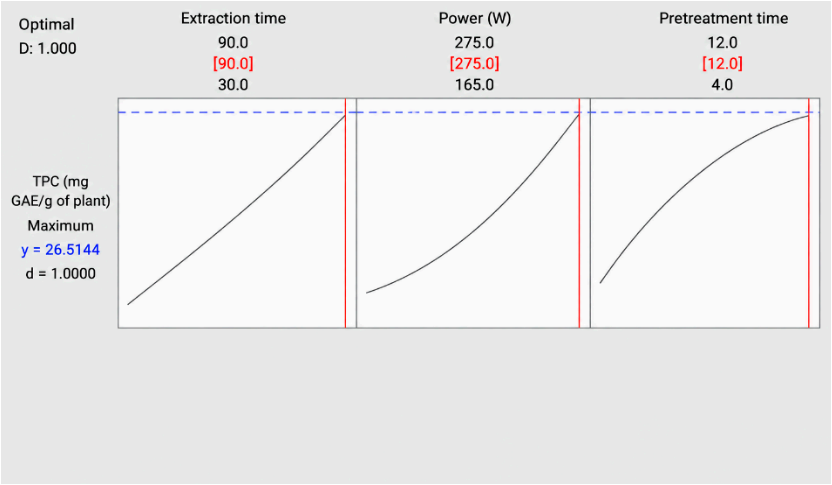

Supplementary Figure S2. Response optimizer plot for TPC (mg GAE/g of plant) of *P. balansae*, showing the effects of extraction time, power (W), and pretreatment time and the selected optimal settings (red vertical lines) with composite desirability D = 1.0000.

LORENTZII

Response Surface Regression: TPC (mg GAE/ g of planta) versus Extraction time; Power (W); Pretreatment time

Coded Coefficients

Term	Coef	SE Coef	T-Value	P-Value	VIF
Constant	3.733	0.206	18.13	0.000	
Extraction time	0.814	0.126	6.46	0.001	1.00
Power (W)	0.354	0.126	2.81	0.038	1.00
Pretreatment time	0.115	0.126	0.91	0.405	1.00
Extraction time*Extraction time	0.019	0.186	0.10	0.921	1.01
Power (W)*Power (W)	0.216	0.186	1.17	0.296	1.01
Pretreatment time*Pretreatment time	-0.281	0.186	-1.51	0.191	1.01
Extraction time*Power (W)	0.601	0.178	3.37	0.020	1.00
Extraction time*Pretreatment time	1.188	0.178	6.66	0.001	1.00
Power (W)*Pretreatment time	0.640	0.178	3.59	0.016	1.00

Supplementary Table S13. Coded coefficients of the response surface regression model for total phenolic content (TPC, mg GAE/g of plant) of *P. lorentzii* as a function of extraction time, power (W), and pretreatment time.

Model Summary

S	R2	R2(aj)	R2(pred)
0.356696	96.09%	89.06%	60.38%

Supplementary Table S14. Model summary for the response surface regression of TPC (mg GAE/g of plant) of *P. lorentzii*, including S, R^2, adjusted R^2, and predicted R^2.

Analysis of Variance

Source	DF	Adj SS	Adj MS	F-Value	P-Value
Model	9	15.6437	1.73819	13.66	0.005
Linear	3	6.4131	2.13771	16.80	0.005
Extraction time	1	5.3039	5.30394	41.69	0.001
Power (W)	1	1.0042	1.00424	7.89	0.038
Pretreatment time	1	0.1050	0.10496	0.82	0.405
Square	3	0.5040	0.16798	1.32	0.366
Extraction time*Extraction time	1	0.0014	0.00139	0.01	0.921
Power (W)*Power (W)	1	0.1729	0.17294	1.36	0.296
Pretreatment time*Pretreatment time	1	0.2914	0.29136	2.29	0.191
2-Way Interaction	3	8.7267	2.90888	22.86	0.002
Extraction time*Power (W)	1	1.4429	1.44285	11.34	0.020
Extraction time*Pretreatment time	1	5.6466	5.64662	44.38	0.001
Power (W)*Pretreatment time	1	1.6372	1.63718	12.87	0.016
Error	5	0.6362	0.12723		
Lack-of-Fit	3	0.3650	0.12167	0.90	0.565
Pure Errorr	2	0.2711	0.13557	*	*
Total	14	16.2799			

Supplementary Table S15. Analysis of variance (ANOVA) for the response surface regression model of TPC (mg GAE/g of plant) of *P. lorentzii*, including linear, quadratic, and two-way interaction terms, lack-of-fit, and pure error.

Regression Equation in Uncoded Units

TPC (mg GAE/ g of planta) = 17.55 - 0.1347 Extraction time - 0.0701 Power (W)
- 0.924 Pretreatment time
+ 0.000022 Extraction time * Extraction time
+ 0.000072 Power (W)* Power (W)
- 0.0176 Pretreatment time * Pretreatment time
+ 0.000364 Extraction time * Power (W)
+ 0.00990 Extraction time * Pretreatment time + 0.002908 Power (W)* Pretreatment time

Supplementary Equation S3. Regression equation in uncoded units for TPC (mg GAE/g of plant) of *P. lorentzii* as a function of extraction time, power (W), and pretreatment time.

LORENTZII

Response Optimization: TPC (mg GAE/ g of planta)

Parameters

Response	Goal	Lower	Target	Upper	Weight	Importance
TPC (mg GAE/ g of plant)	Maximum	1.62136	6.01543		1	1

Supplementary Table S16. Response optimization parameters for maximizing TPC (mg GAE/g of plant) of *P. lorentzii*.

Solution

Solution	Extraction time	Power (W)	Pretreatment time	TPC (mg GAE/ g of plant) Fit	Desirability Composite
1	90	275	12	7.39980	1

Supplementary Table S17. Response optimization solution for *P. lorentzii*, showing the selected extraction time, power, pretreatment time, fitted TPC, and composite desirability.

Multiple Response Prediction

Variable	Setting
Extraction time	90
Power (W)	275
Pretreatment time	12

Response	Fit	SE of the Ajusted	95% CI	95% PI
TPC (mg GAE/ g of planta)	7.400	0.421	(6.317; 8.483)	(5.981; 8.819)

Supplementary Table S18. Multiple response prediction for TPC (mg GAE/g of plant) of *P. lorentzii* at the selected extraction time, power, and pretreatment time, including fitted value, standard error of fit, 95% confidence interval, and 95% prediction interval.

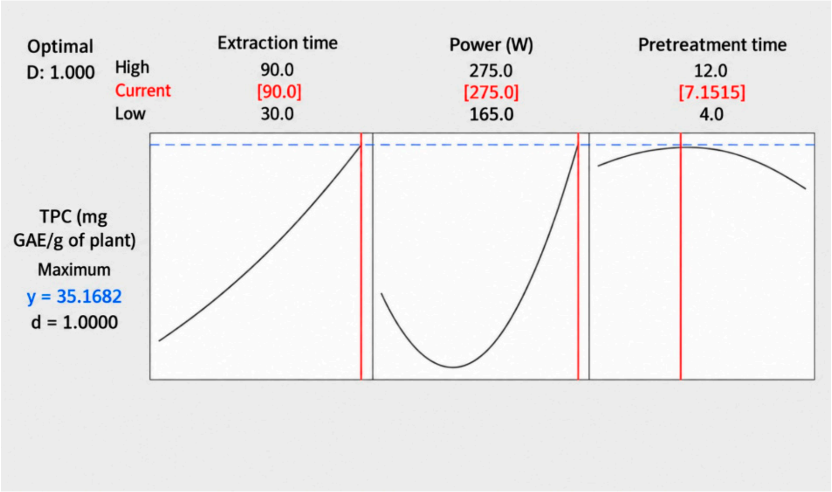

Supplementary Figure S3. Response optimizer plot for TPC (mg GAE/g of plant) of *P. lorentzii*, showing the effects of extraction time, power (W), and pretreatment time and the selected optimal settings (red vertical lines) with composite desirability D = 1.0000.
